# The use and usefulness of the Peninsula Health Falls Risk Assessment Tool (PHFRAT) process in residential aged care: a mixed methods study across 25 aged care facilities

**DOI:** 10.1186/s12877-024-05462-8

**Published:** 2024-10-24

**Authors:** Crisostomo Ibarra Mercado, Isabelle Meulenbroeks, Guogui Huang, Nasir Wabe, Karla Seaman, Joanna Clive, Johanna Westbrook

**Affiliations:** Centre for Health Systems and Safety Research, Australian Institute of Health Innovation, Macquarie University, Level 6, 75 Talavera Rd, 2113 Macquarie Park, NSW Australia

**Keywords:** Peninsula Health Falls Risk Assessment Tool, Fall prevention, Process map, Residential aged care, Falls

## Abstract

**Background:**

Falls remain a persistent problem in residential aged care (RAC) facilities. Fall screening and assessment tools such as the Peninsula Health Falls Risk Assessment Tool (PHFRAT) are widely used to inform falls risk and guide fall prevention interventions. However, it is unclear how it is used in practice and whether clinicians believe it supports resident care. This study aimed to measure the extent of use of PHFRAT to understand clinicians’ perceptions of its value and usefulness.

**Methods:**

This mixed method study involved an analysis of PHFRAT assessment from 25 RAC facilities in New South Wales, Australia, and interviews with seven RAC staff about how PHFRAT information is used in practice. In the quantitative component, descriptive statistics were applied to PHFRAT data to summarise how RAC staff use the PHFRAT including the completeness and content of the three parts. In the qualitative component, thematic analysis techniques were applied to interview data.

**Results:**

The sample included 215 RAC residents with 703 PHFRATs, of which 617 documented fall prevention interventions. Among these 617 PHFRATs, 593 (96.1%) included strategies related to staff assistance and 283 (45.9%) recorded strategies related to device provision. While nearly all residents (96.74%) received at least one PHFRAT assessment over the study period, many PHFRAT assessments were incomplete (part 1: 11.5% of information missing; part 2: 10.8%; part 3: 17.1%). There were few variations in fall interventions prescribed to individual residents by their fall risk level. Interviews with RAC staff indicated that PHFRAT assessments are the responsibility of registered nurses with limited input from other staff or residents. While the structured process was viewed positively in guiding risk assessment and intervention assessment, a lack of input from others prevented strategies from being tailored to residents’ specific needs and preferences. A shortage of resources, lack of communication, and limited staff education were identified as the main barriers to PHFRAT guideline implementation.

**Conclusion:**

The PHFRAT provides a useful structure for clinicians to assess falls risk factors and plan falls prevention strategies. In the future, increased multidisciplinary input into fall prevention strategy development may improve the comprehensiveness of fall prevention plans.

**Supplementary Information:**

The online version contains supplementary material available at 10.1186/s12877-024-05462-8.

## Introduction

Falls in residential aged care (RAC) are very worrying to residents, families, clinicians, and RAC providers. Almost 50% of RAC residents fall more than once a year [1–4], with a crude incidence rate of 7.14 falls per 1000 resident days [5]. Falls can lead to severe clinical consequences for RAC residents, including injuries, multisite pain, decreased quality of life, disability, and even death [6]. The high prevalence and severe consequences of falls in RAC present challenges for RAC service providers and clinicians in identifying residents most at risk and the importance on resourcing effective fall prevention interventions.

To effectively manage and prevent falls, facilities often use fall risk screening and assessment tools to predict residents at risk of falling [7, 8]. Several fall risk assessment tools have been created but few have undergone proper evaluation, making the effectiveness of certain fall risk assessment tools in RAC uncertain [7, 9]. The Peninsula Health Falls Risk Assessment Tool (PHFRAT) is a validated tool to screen, assess fall risk, and optimise fall prevention management [9]. Developed in 1999 by Peninsula Health in Victoria, Australia, this tool has been widely used by clinicians in Australian RAC facilities, and shown to have a high compliance rate with one study showing more than 90% of residents having a completed assessment within 24 h of admission [10]. The PHFRAT guidelines comprise three parts: part 1, categorising residents’ fall risk levels based on resident profile information as low, medium, or high risk; part 2, evaluating the risk factors of falls; and part 3, documenting the fall prevention interventions required to minimise the risk for each problem identified in part 1 and part 2 (Appendix 1) [7]. The use of PHFRAT in RAC is designed to assist clinicians and RAC service providers to better allocate resources to residents most in need and to ensure those at high fall risk receive appropriate and timely fall prevention interventions [11–13].

However, the PHFRAT also has limitations in fall prediction and prevention. A small prospective cohort study conducted by Barker et al. [9] questioned the predictive validity of PHFRAT (along with three other fall risk assessment tools) in RAC, and reported poor performance on a range of measures such as reliability and evaluative and discriminative validity testing. Similarly, our recent study of 5,888 residents demonstrated PHFRAT to have poor predictive performance, with a sensitivity of 26.0%, specificity of 88.8%, and receiver operating characteristic curve area under the curve (ROC AUC) of 0.57 [14]. The poor predictiveness of the PHFRAT raises concerns about the tool including whether some residents who fail to be identified as at risk of falls may be missing out on necessary fall prevention interventions.

While these studies have raised questions about the performance of the PHFRAT, little is known about how PHFRAT guidelines and recommended actions are implemented in daily RAC practice. The PHFRAT provides a standardised framework for evaluating fall risk, ensuring that all relevant factors are systematically considered rather than relying on ad-hoc judgments. The tool is also highly feasible, typically taking less than three minutes to complete, with high 24-hour compliance by RAC staff [10]. Its design, with three parts focusing on fall status evaluation, risk factor identification, and strategies documentation, respectively, aligns with recommendations from previous studies [15–17]. Consequently, the PHFRAT was recommended by the Australian Commission on Safety and Quality in Healthcare in 2009 for use in Australian RAC facilities [18]. Therefore, it is possible that while the tool may not be accurate at predicting falls risk, clinicians may find it useful in resident care, particularly in ensuring comprehensiveness when clinicians assess potential falls risk and providing necessary documentation and accountability of clinicians’ assessment and intervention process.

While the 2009 Australian guidelines recommended the use of risk stratification tools such as the PHFRAT, recent international guidelines advise against this approach [11, 19, 20]. Current evidence suggests that all residents should be considered high risk for falls, which contrasts with the stratification method inherent in PHFRAT [11, 19, 20]. This shift in perspective necessitates a re-evaluation of the tool’s applicability in contemporary practice. This study aimed to explore the use of PHFRAT in RAC using quantitative and qualitative methods to comprehensively understand the extent of use of the PHFRAT and its various components and to understand clinicians’ perceptions of its value and usefulness.

## Methodology

### Design

This was a mixed methods study comprised of two components using a two-phase explanatory sequential design [21]. The first phase included a quantitative component based on a descriptive analysis of linked datasets from 25 RAC facilities administered by a large not-for-profit RAC service provider in New South Wales, Australia. Data findings from the first phase were used to inform the design of the second component (qualitative research), specifically, the interview design. Interviews were conducted with RAC staff about how PHFRAT information is used. However, the qualitative interviews in the second phase went beyond the scope of the quantitative component by exploring staff’s personal reflection on PHFRAT use, limitations of this scale, and suggestions for future improvement, to obtain a more detailed understanding of the user experience of PHFRAT in RAC. Drawing on quantitative and qualitative components together permitted exploration of how clinicians implement the PHFRAT guidelines and if and how the PHRAT supports fall prevention practices. This study was approved by the Macquarie University Ethics Committee (Ref: 52019614412614). This study involved aged care staff and did not directly involve older people.

### Analysis of linked datasets

Linked data which contained resident profiles and PHFRAT data for 25 RAC facilities were used. The resident profile dataset provides information of residents’ demographic characteristics (e.g., age, gender), health conditions at admission (e.g., dementia, diabetes), and admission-related information (e.g., admission type, facility), while the PHFRAT dataset provides information on, but not limited to, date and time when PHFRAT was conducted, fall risk status, fall risk factors, and fall prevention interventions that residents received. Data related to all falls in 2019 were assessed. The analysis was limited to permanent residents with a length of stay of at least 30 days because PHFRAT assessments are generally unavailable for residents receiving interim care and are not sufficiently followed up for residents receiving respite care. This yielded a sample of 215 RAC residents with 703 PHFRATs for analysis.

**Fig. 1:**
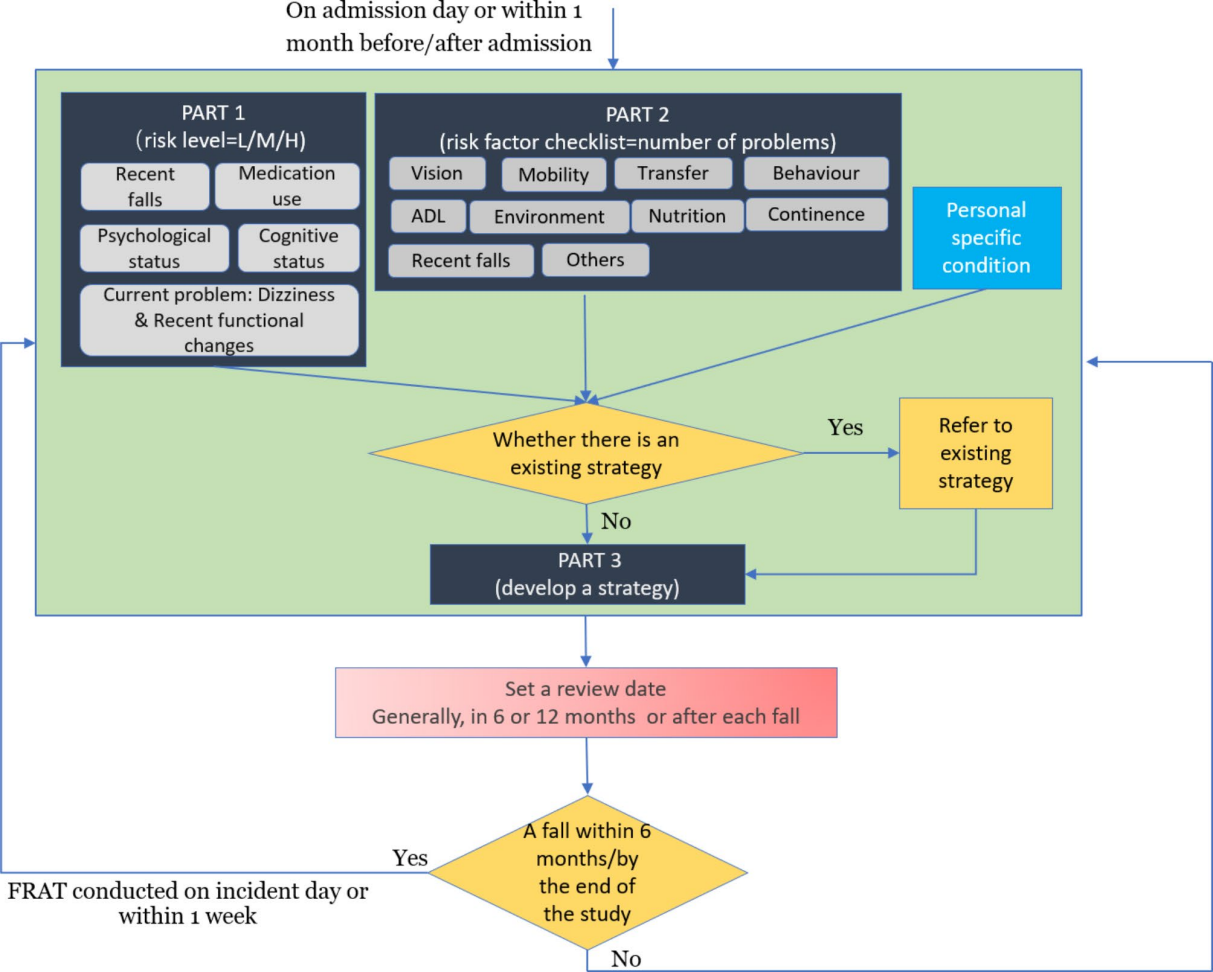
PHFRAT guidelines flowchart (How PHFRAT is accurately done) *Note: H/M/L refers to high*,* medium*,* low and ADL refers to activities of daily living*

A process map (Fig. 1) outlining the PHFRAT guidelines and workflow associated with completing and actioning a PHFRAT was prepared [22]. Figure 1 describes the three steps of undertaking a PHFRAT assessment: assess resident’s level of fall risk (part 1), identify fall risk factors (part 2), and develop fall prevention interventions (part 3). In part 1, PHFRAT guides an assessment of fall risk level by considering the resident’s number of recent falls, medication use, and cognitive status as well as their ability to safely mobilise and any history of dizziness/postural hypotension. These elements are scored out of 20 to determine the falls risk. A total score of 5–11 is considered a low fall risk; 12–15 medium risk; and 16–20 high risk. In part 2 of PHRAT, nine fall risk factors including vision, mobility, continence, and environment are assessed. In part 3, possible fall prevention strategies are considered, selected, and documented (e.g., resident likes to have wine with their dinner; staff to assist resident in walking to the dining room and back to the room using a four-wheel walker frame) (Appendix 1). The regularity of completion of PHFRAT assessment varies depending on the aged care provider’s policy, however, it is recommended that all residents (permanent and respite) should have a completed PHFRAT within 24 h of admission and should be re-evaluated three monthly and yearly, or after every fall, whichever occurs first [22].

Descriptive statistics were calculated to populate Fig. 1 to quantitatively map the application of the PHFRAT (i.e., number of assessments, health conditions, personal specific conditions, interventions, and repeat assessments). Since the information on interventions is largely text-based, one researcher (GH) with expertise in fall prevention interventions manually reviewed all the records of interventions documented in the 703 PHFRAT assessments. Based on published guidelines and literature on fall prevention interventions [19, 23–25], GH categorised interventions into ten broad groups and 31 further specific types by their content using an inductive approach [26]. The categorisation methods were discussed and confirmed with the research team to gain consensus and then applied to the data. The findings were reviewed and improved through team meetings. The quantitative analyses were conducted using Microsoft Excel and Stata version 17.0.

### Staff interviews

Using the PHFRAT guidelines flowchart (Fig. 1) and findings from the analysis of quantitative PHFRAT data, a 20-minute structured interview schedule (Appendix 2) was designed. The interviews were conducted to explore the differences between PHFRAT guidelines (Fig. 1) and how PHFRAT is done in everyday clinical work; for example, questions asked if the process map accurately describes how PHFRAT assessments are done in the facility and if there are procedures missing. Then quantitative descriptive results from the PHFRAT analysis which demonstrated how clinicians use the PHFRAT in everyday clinical work were shared with interview participants. The interview schedule was piloted prior to use with two clinicians.

Clinicians with experience using the PHFRAT who were currently working at the participating aged care provider were eligible to participate, including managers, nurses, workplace trainers, and allied health workers. In Australian RAC, managers and workplace trainers are typically nurses who have been promoted to administrative, managerial, and educational roles. Participants were interviewed online or face-to-face at their choosing. Participants were recruited through the participating aged care provider using snowballing strategies. Written consent was collected from all participants, and all received a $25 gift voucher for participation.

Interviews were conducted by one researcher with no prior experience conducting qualitative research (CM). However, they were supported by and received feedback from researchers with experience interviewing healthcare staff (KS, IM). Interviews with clinicians were audio recorded and transcribed verbatim. Transcripts were manually checked for accuracy and deidentified prior to analysis. Interviews were thematically analysed using six steps: familiarisation, coding, initial themes, developing themes, refining themes, and writing up [27]. A deductive approach was applied using the cyclical nursing process model which is routinely used by nurses to critically think and systematically solve nursing problems or situations in everyday clinical work. This model includes assessment, diagnosis, planning, implementation, and evaluation [28–30]. Communication is added as a sixth step and highlighted as an important element for safe and effective nursing practice [31, 32]. The final coding structure involving assessment, planning, implementation, evaluation, and communication (i.e., APIE-C) was selected to help map the roles and responsibilities of clinicians in completing PHFRAT and reflect on the PHFRAT’s value and usefulness, and application in RAC facilities (evaluation). Two researchers (IM and CM) independently coded 40% of the transcripts then met to discuss codes and develop a coding strategy. The remainder of the interviews were coded by CM and changes to the coding structure discussed in team meetings. Both coders have experience working as health professionals in aged care and had used the PHFRAT clinically, previously (IM, CM).

## Results

### Results from the analysis of the PHFRAT data

#### RAC residents with a PHFRAT assessment

The sample comprised 215 RAC residents who had a total of 703 PHFRAT assessments. Approximately two-thirds of the participants were female (66.05%), aged 85 years and older (56.75%), and born in Australia (67.91%) (Table 1). The median length of stay of residents was 159 (interquartile range [IQR]: 95–272) days and more than half of them (54.88%) had previously experienced falls. Overall, 46.05% (*n* = 99) of residents had a diagnosis of dementia, 46.05% (*n* = 99) had depression and 43.26% (*n* = 93) had anxiety, while nearly all the residents (96.74%) received at least one PHFRAT assessment over the study period (Table 1).

**Table 1:** Baseline characteristics of the 215 RACF residents

Characteristics		*N* (%)	%
Sex	Male	73	33.95
	Female	142	66.05
Age	65–74	17	7.91
	75–84	76	35.35
	85–94	109	50.70
	≥ 95	13	6.05
Fall status	Faller	118	54.88
	Non-faller	97	45.12
Number of PHFRAT per resident	0	7	3.26
	1	79	36.74
	2–3	56	26.05
	4–5	43	20.00
	≥ 6	30	13.95
Length of stay (days), median (IQR)		159 (95–272)	
Health conditions			
Dementia		99	46.05
Depression		99	46.05
Anxiety		93	43.26
Cerebrovascular accident		60	27.91
Diabetes mellitus		61	28.37
Visual impairment		43	20.00
Delirium		33	15.35
Parkinson’s disease		12	5.58
**Total**		215	100

#### How PHFRAT is done in everyday clinical work

**Fig. 2:**
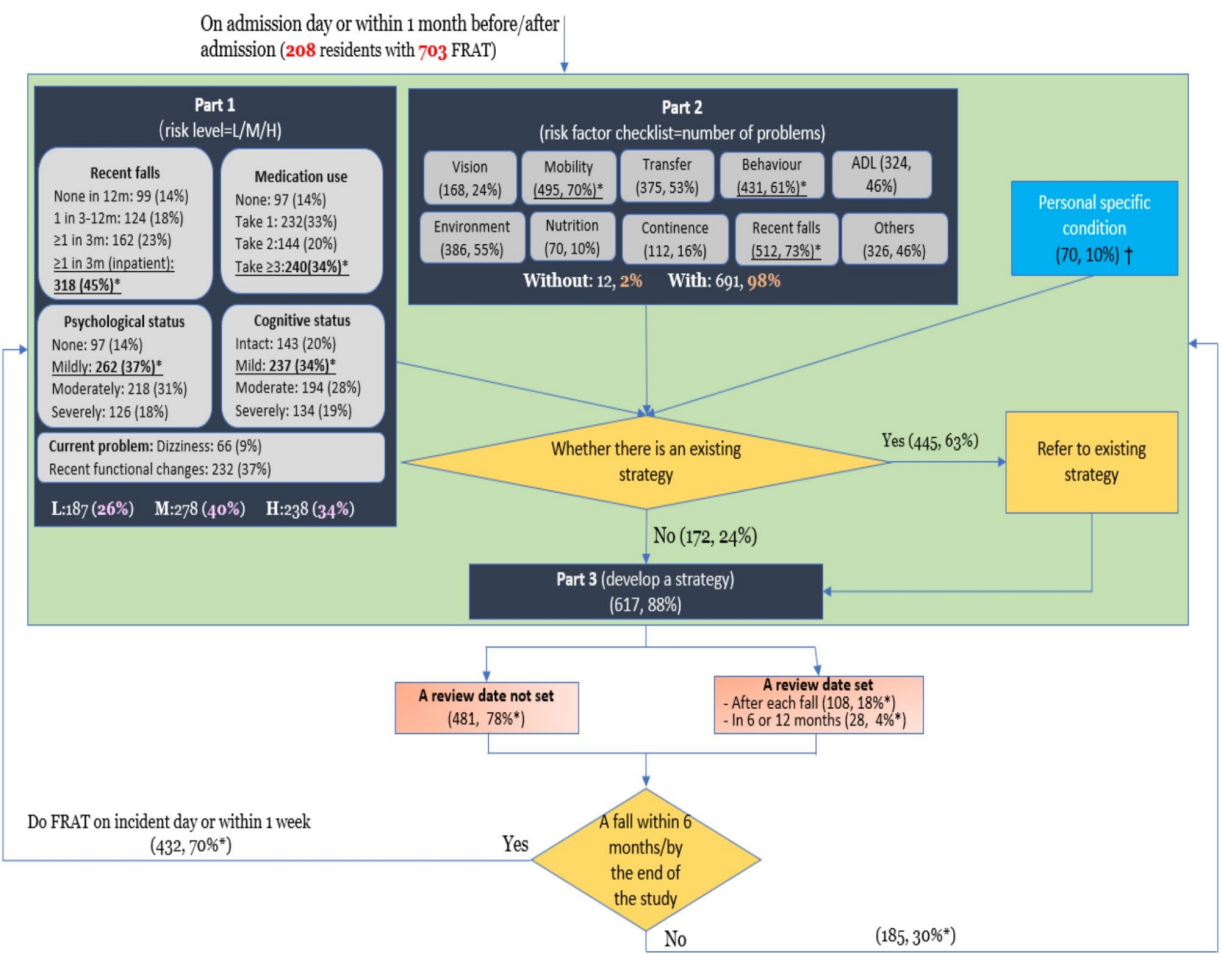
How *PHFRAT is done in everyday clinical work* Note: *,* using 617 as denominator; †*,* personal situations considered in fall prevention strategies*,* such as family opinion*,* daily routine*,* individual habits; H/M/L refers to High/Medium/Low. These three levels correspond to a score of part 1 ranging from 16 to 20*,* 15 − 12 and 5–11*,* respectively*

Figure 2 presents the flowchart of how RAC staff use the PHFRAT in everyday practice showing if and how the procedures outlined in Fig. 1 were followed. As shown, among the 703 PHFRATs audited, 326 (46%) were conducted within one month of admission. In part 1, 187 residents (26%) were assessed as low fall risk, 278 (40%) as medium risk, and 238 (34%) as high risk of falling.

In part 2, 691 (98%) PHFRATs identified at least one fall risk factor, among which recent fall history (512, 73%), mobility-related problem (495, 70%), and behaviour-related problem (431, 61%) were the most common. On average, 4.6 fall risk factors were identified per PHFRAT. The number of risk factors per PHFRAT was similar by gender (4.6 for females and 4.5 for males) but increased as fall risk level rose (3.1 for low risk level, 4.3 for medium risk level, and 6 for high risk level).

In part 3, 617 (88%) PHFRATs documented fall prevention interventions or strategies. On average, 3.8 interventions were documented per PHFRAT. The mean number of interventions were similar by gender (3.9 for males and 3.8 for females) but increased as fall risk level increased (3.6 for low risk level, 3.7 for medium risk level, and 4.1 for high risk level).

Among those with fall prevention interventions documented, 136 (22%) had a review date set, and 432 (70%) were followed by another PHFRAT assessment ahead of the scheduled review date due to the occurrence of a fall.

#### Fall intervention types

Fall interventions documented in part 3 were classified into 31 specific types under ten broad groups. Three of the ten broad interventions were focused on device provision (i.e., providing devices for mobility improvement, providing devices for fall prevention and providing devices for reducing fall-related injuries), and seven focused on staff providing assistance (i.e., staff’s direct help, staff’s close supervision, encouraging use of staff assistance and device, device maintenance and quality check, keeping the environment safe, providing nutrition, and other measures) (Table 2).

Among the 617 PHFRATs documenting fall prevention interventions, 283 (45.9%) recorded strategies related to device provision, of which providing fall prevention devices (e.g., installing call bells/sensors/sensor mats) was the most common (38.7%). In addition, 593 (96.1%) PHFRATs’ strategies related to staff assistance (Table 2) were documented, of which staff providing direct help was the most common (62.9%). The most frequently used specific type of fall prevention intervention was direct help from staff (62.9%), followed by close staff supervision (39.9%), check the condition/function of caller/buzzer (24.8%), and monitor resident clothing (e.g., check resident’s clothes to ensure it’s not loose as it can get tangled up with doors or furniture) (24.5%). Providing a hip protector (2.4%), physiotherapy referral (4.2%), anti-slip shoes/socks (4.9%), and devices for mobility improvement (4.9%) were the least commonly used fall prevention interventions.

The types of interventions prescribed were similar for residents with a low, medium, and high risk of falls, all with the top three common type of interventions being: direct staff assistance, device maintanence and quality check, and providing fall prevention device, regardless of the risk level. This suggests that commonly used fall prevention interventions did not appear sensitive to changes in fall risk level (Appendix 3).

**Table 2:** Different types of fall interventions and their prevalence

	Broad group	Specific type
Device provision (45.9%)	1-Mobility improvement (4.9%)	(1) provide devices for mobility (e.g., walker) (4.9%)
	2-Fall prevention (38.7%)	(2) provide chair/bed rail (10.2%)
		(3) provide anti-slip shoes/sock (4.9%)
		(4) provide caller (5.8%)
		(5) install sensor (12.5%)
		(6) install sensor mat (13.1%)
	3-Reduce fall-related injury (15.4%)	(7) install low-low bed (14.9%)
		(8) install crash mat (13.5%)
		(9) provide hip protector (2.4%)
Staff assistance (96.1%)	4-Direct help (62.9%)	(10) direct assistance (62.9%)
	5-Supervision (39.9%)	(11) close supervision (39.9%)
	6-Encourage to use staff help or equipment (36.1%)	(12) encourage to use staff’s help (18.6%)
		(13) encourage to use mobility devices (15.4%)
		(14) encourage to use callers (16.0%)
	7-Device maintenance and quality check (47.0%)	(15) check mobility devices (13.8%)
		(16) check bed conditions (12.6%)
		(17) check vision devices (6.8%)
		(18) check callers (24.8%)
		(19) check clothing (24.5%)
		(20) check mats (6.5%)
		(21) check sensors (6.8%)
		(22) check toilet (6.2%)
	8-Environment (17.3%)	(23) keep environment safe (17.3%)
	9-Nutrition (15.1%)	(24) provide nutrition (15.1%)
	10-Other (33.5%)	(25) experience sharing (7.1%)
		(26) provide fall education (12.8%)
		(27) encourage social interaction (11.7%)
		(28) health status monitoring (6.6%)
		(29) pain management (7.6%)
		(30) physiotherapy referral (4.2%)
		(31) others-personalised (e.g., monitoring alcohol consumption) (15.2%)

### Interview results

Seven interviews were conducted (three online and four face-to-face). Occupational information of the seven participants are presented in Table 3. Interviews lasted between 10 and 28 min (interview length: mean 18 min, median 15 min).

**Table 3:** Occupational type of interviewees

Professional group			Number of interviews
Care Manager			2 (I01, I03)
Physiotherapist			1 (I04)
Quality and Compliance Manager			1 (I02)
Registered Nurse			2 (I05, I06)
Workplace Trainer			1 (I07)
**Total**			**7**

**Fig. 3:**
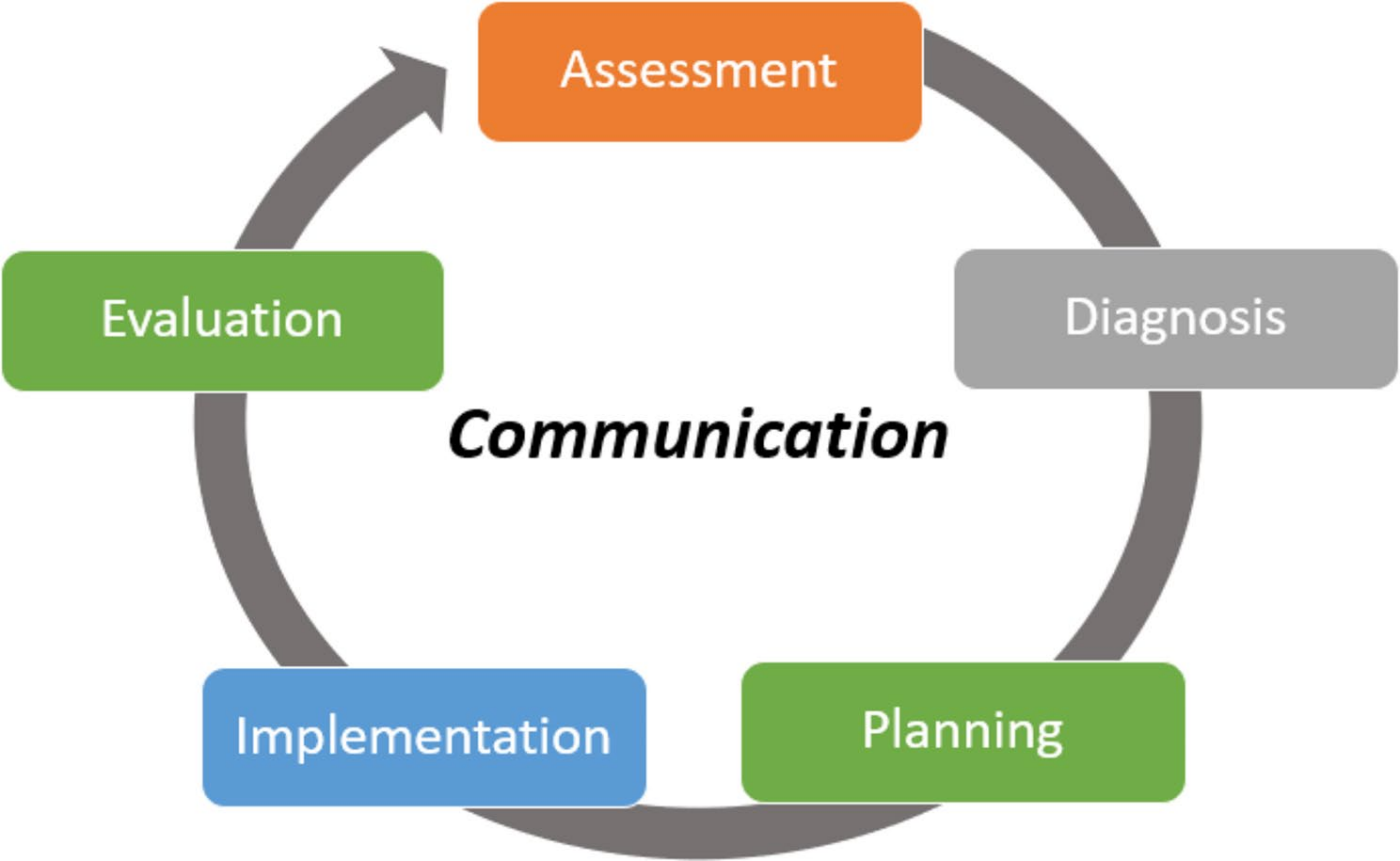
The Assessment, Diagnosis, Planning, Implementation, Evaluation and Communication (ADPIE-C) coding strategy

Two themes were created with subthemes using the APIE-C coding structure (Fig. 3). In theme 1, *PHFRAT: Nurse led assessment and diagnosis of fall risk*, participants described how they implemented and used the PHFRAT in their everyday clinical work including how the assessment was completed and results communicated. In theme 2, *Evaluation of the PHFRAT*, participants reflected on the gaps in practice, the practicality of the assessment and ways in which the PHFRAT could be improved.

### Theme 1: PHFRAT: nurse-led assessment and diagnosis of fall risk

Participants reported that all the components of the assessment (parts 1, 2, and 3) are completed by registered nurses (RNs) at the facilities at admission, and every 3–12 months or after a fall. Participants reported that other multidisciplinary team members were sometimes indirectly involved in PHFRAT re-assessment after a fall

“So every time someone had a fall, we put the review [the PHFRAT], and with the physios as well” (I05, RN).

“Yes, so if there is a fall or something we find out the reason behind or we try to observe or … we refer to the doctors or the OTs.” (I07, workplace trainer).

Participants reported that the structure of the PHFRAT guided nurses through a person-centred fall risk assessment process and the diagnosis of fall risk (i.e., low, medium, or high risk) as the structure of the PHFRAT encourages assessors to consider the local circumstance and the resident’s needs and wants. The uniform and structured diagnosis process allowed nurses to tailor falls prevention interventions and care intensity to residents who were at higher risk of falls.

“If we have two residents with the same vision mobility and transfer issues, but one’s a low risk and one’s having lots of risks [due to social factors], they would have different strategies.”( I06, RN).

### Theme 2: evaluation of the PHFRAT

When presented with the quantitative results, participants reflected on the gaps in current practice and discussed methods by which the PHFRAT assessment could be improved. Missing or delayed elements of the PHFRAT assessments, including missing re-assessments or an absence of or limited interventions, and the same number and type of interventions regardless of risk category were reportedly caused by poor staff education, inadequate resources—including facility equipment and insufficient personnel, and the recent clinical management system software update was not well-suited to their current workflow.

“*…*So, the PHFRAT is only evaluated if there’s another fall… And then we we update our care plans every year and then evaluate it every three months. But I guess the hindrance of that is the number of resources of registered nurses that we have. And there’s a tendency that it will affect the quality of the evaluation.” (I01, Care Manager).

“I think it comes down to the RNs not recognising, or, not even looking at the risk level and risk factor checklist in detail to identify what could be the personal strategy for this resident and knowing their personal specific condition.” (I02, Quality and Compliance Manager).

In addition, participants did not believe that all the information entered in the PHFRAT was accurate and up-to-date. One participant stated that PHFRAT information is only “as good as the clinician’s experience.”

“… I do not believe so. At times, yeah. It all depends on the RNs expertise, and the physio – how they have initiated the mobility assessment, and it all depends on how much review has been carried out at that time, during the fall.” (I02, Quality and Compliance Manager).

In general, participants discussed PHFRAT as being “holistic” (I04, Physiotherapist) as it guides the assessor to consider special conditions including resident and family preferences and a “wonderful tool to have” (I03, Care manager). However, some participants doubted its value and use in RAC facilities, partly due to missing information of fall prevention/intervention in Part 3.

“… It’s just another form we have to fill out when they have a fall. It’s just a tick box, I feel like sometimes.” (I06, RN).

“You can’t just rely on the information in the FRAT …. it takes a little bit more digging in the resident’s information.” (I03, Care Manager).

Most participants indentified some ways in which the PHFRAT could be improved. Suggestions included a checklist of strategies to include to assist RNs in their decision making to complete the assessment, especially for those who are new to their roles. Having a PHFRAT form with mandatory fields to complete within the IT system was also emphasised to be an imortant forcing function. Lastly, greater multidiciplinary involvement in the conduct and completion of the PHFRAT assessment and implementation of strategies was raised as important. Participants firmly believed that preventing a resident from falling is “everyone’s responsibility.” (I05, RN).

### Theme 3: fall risk communication

Participants reported that the outcomes of the PHFRAT, including fall risk prevention strategies, were inconsistently handed over to the rest of the care team. Some participants stated that only the resident changes such as a change in the assessed fall risk level (i.e., low to medium or high) and intervention changes are communicated, not the entire PHFRAT information.

“*…* No. I would say no. So, it would be more like if we need assistance from the physio or something. We would contact them, usually verbally or by email. Usually, the care staff only really know about, maybe if we’ve got interventions after the fall… Verbally in the handover just say, okay, room … has a sensor beam because they keep falling.” (I06, RN).

In the quantitative analysis, communication of fall risk prevention strategies was incomplete; 12% of PHFRATs did not have documented fall prevention strategies. Participants who reflected on this expressed that the gap in communication within the documentation may be due to strong personal relationships between care staff and the resident and reduced focus on fall prevention interventions for residents with low falls risk.

“*…*And I think as well probably the capacity to complete a strategy. There are times I’ve seen the staff are delivering the strategies, especially the care staff because they know more what the resident likes or dislikes… But I guess, it’s not shared to the whole team, or it’s not reflecting on the care plan… or it’s not reflecting on the PHFRAT as well.” (I01, Care Manager).

“I guess if there’s a low risk, or the resident hardly falls, there wouldn’t be anything. … it’s because they don’t fall, they haven’t had a fall so it hasn’t been triggered as an issue.“ (I06, RN).

## Discussion

Our study showed that PHFRAT is widely used in Australian RAC facilities, with nearly all residents (96.74%) receiving at least one PHFRAT assessment over the study period. However, many PHFRAT assessments were incomplete (part 1: 11.5% had information missing; part 2: 10.8% missing; part 3: 17.1% missing), and where interventions were identified, there was little variation in the type of interventions prescribed to individual residents. Although the PHFRATs in this audit were often incomplete, and all had similar information, clinicians reported that they thought the PHFRAT useful and necessary for everyday clinical work in RAC.

Overall, the fall prevention interventions recommended in the PHFRATs in this study lacked variability and predominately included providing close supervision (39.9%) and check call bells (24.8%). The most common recommendations in the PHFRAT contrast significant evidence-based falls prevention recommendations in RAC—exercise and multifactorial assessment and management of contributing factors such as cardiovascular health, nutrition, and the environment— [18, 33–36] which were rarely recommended in PHFRATs. For example, while devices such as call bells (24.8%), low-low beds (14.9%), and crash mats (13.5%) were recommended in PHFRATs in this study, evidence of their effectiveness varies. Systematic reviews and international guidelines indicate that some of these interventions may have limited benefit or could even increase the risk of falls [37]. Studies have shown that while sensors and low-low beds can reduce fall-related injuries, they do not significantly reduce (or even increase) the incidence of falls themselves [38, 39]. Conversely, interventions such as medication review and deprescribing, which are not explicitly prompted by the PHFRAT, have been shown to effectively reduce fall risk in RAC settings [40, 41]. Limited multidisciplinary practice in RAC likely explains the lack of variability in evidence-based falls prevention interventions. In our interviews, we found that all PHFRATs were completed by RNs. While many RNs are very skilled in fall prevention, RNs alone should not be expected to be solely responsible for knowing a resident’s fall-related information, nor do they have the capacity to routinely check, review, and update such information for every resident such as interacting, high-risk medications, nutritional status, and balance.

In our study, the number and type of falls prevention strategies recommended by staff in the PHFRAT also did not differ between residents despite being categorised in low, medium, or high risk of falling by the predictive scoring in the PHFRAT. This suggests that the predictive component of the PHFRAT may not be clinically relevant as it is not associated with a change in practice. Falls risk predictive tools have also been criticised internationally for their often poor predictive power, heavy reliance on history of falls limiting their ability to identify first time fallers, and lack of emphasis on modifiable risk factors which could be used to guide falls prevention interventions [9, 14, 42]. As a result of the limited predictive power and practical guidance, international guidelines have recommended against the use of screening tools to predict fall risk in the RAC population indicating that it is not a good use of resources, and that instead all residents should be considered high fall risk [11, 19].

Despite the poor predictive power of the PHFRAT and the observed lack of diversity in fall prevention strategies, the clinicians interviewed in our study reported that they found the PHFRAT useful. We postulate that the clinicians’ perceived benefit of the PHFRAT arises from the PHRAT processes that facilitate the identification of fall risk factors and documentation of proposed actions. Notably, the PHFRAT, unlike some other falls risk assessment tools, highlights modifiable risk factors such as weight, equipment use, clothing, and shoes which appear to be utilised to some extent in suggested fall prevention interventions in this study [9, 42]. Currently, within these facilities, the PHFRAT provides RAC staff with a structured approach to assess and identify fall risk and risk factors (parts 1 and 2), process this information to plan and prescribe fall prevention interventions and use this information as a basis for action (part 3) [43, 44]. Interventions in part 3 of the PHFRAT assessment are then documented in the resident care plan (i.e., mobility care plan). In this way, the PHFRAT acts as a clinical care pathway (assessment, planning, and intervention) rather than just a tool to predict low, medium, and high risk of falls.

These results have real world implications. Recent international guidelines have recommended against the use of screening tools to predict fall risk in a RACF population as they are not a good use of resources, and that instead all residents should be considered high fall risk [11, 19]. However, our study suggests that fall screening tools such as the PHFRAT may still be useful to clinicians, not as a predictive tool but as a fall prevention clinical pathway. The routine assessment, planning, and intervention—such as the steps facilitated in the PHFRAT—are useful for everyday clinical work [19] and support clinicians to systematically solve problems and facilitate continuity of care and resident safety. The clinical pathway facilitated by the PHFRAT could be further enhanced by focusing on modifiable risk factors such as physical function and medications, rather than falls history. Additionally, in the future, having improved multidisciplinary involvement, which importantly incorporates residents’ preferences, may enhance the effectiveness of fall prevention strategies even in resource constrained environments in the future [45].

## Strengths and limitations of the study

The strengths of our study lie in the methodology (i.e., mixed method study) utilising both quantitative data representing existing practice with qualitative data from clinicians to gain an integrated understanding of their views and work practices [46]. The study has some limitations. First the qualitative component had a small sample size, which partly reflects the difficulty in aged care staff being released from their roles to participate given staff shortages in the sector. Extensive recruitment strategies were employed to recruit participants including the addition of a gift voucher as an incentive, extension of the recruitment window to four months, and presence on-site. Despite these efforts, no further participants consented to participate due to the COVID-19 pandemic and work demand challenges. In addition, interview lengths were short as participants wanted to return to routine work activities as soon as possible. However, analysis of the interviews demonstrated that data saturation may have been reached at seven interviews. It is also unknown whether the demographics of the participants reflects the people who use the PHFRAT at the provider as these data are not collected. Moreover, the snowball sampling approach used also cannot guarantee the representativeness of the sample in the qualitative interviews. Second, the coding of fall prevention interventions in our quantitative analysis of the PHFRATs was dependent on word choice entered into the records.

## Conclusion

This study highlights the structured approach of the PHFRAT as beneficial for guiding clinicians through assessment and intervention planning in RAC settings. However, to align with current evidence-based practices, it is crucial to integrate more individualised and person-centred fall prevention strategies, moving beyond risk stratification. By involving multidisciplinary input and considering evidence-based interventions, the effectiveness of fall prevention measures can be significantly enhanced. Future research should focus on refining the PHFRAT to incorporate these elements and better meet the needs of the RAC population.

## Electronic supplementary material

Below is the link to the electronic supplementary material.

Supplementary Material 1

## Data Availability

All data generated or analysed during this study are included in this published article and its supplementary information files.

## References

[1] Vlaeyen E, Coussement J, Leysens G, Van der Elst E, Delbaere K, Cambier D, Denhaerynck K, Goemaere S, Wertelaers A, Dobbels F. Characteristics and effectiveness of fall prevention programs in nursing homes: a systematic review and meta-analysis of randomized controlled trials. J Am Geriatr Soc. 2015;63(2):211–21. 25641225 10.1111/jgs.13254

[2] Rubenstein LZ, Josephson KR. The epidemiology of falls and syncope. Clin Geriatr Med. 2002;18(2):141–58. 12180240 10.1016/s0749-0690(02)00002-2

[3] Nyberg L, Gustafson Y, Janson A, Sandman P-O, Eriksson S. Incidence of falls in three different types of geriatric care: a Swedish prospective study. Scand J Soc Med. 1997;25(1):8–13. 9106939 10.1177/140349489702500103

[4] Kannus P, Sievänen H, Palvanen M, Järvinen T, Parkkari J. Prevention of falls and consequent injuries in elderly people. Lancet. 2005;366(9500):1885–93. 16310556 10.1016/S0140-6736(05)67604-0

[5] Wabe N, Seaman KL, Nguyen AD, Siette J, Raban MZ, Hibbert P, Close JC, Lord SR, Westbrook JI. Epidemiology of falls in 25 Australian residential aged care facilities: a retrospective longitudinal cohort study using routinely collected data. Int J Qual Health Care. 2022;34(3):mzac050. 35588391 10.1093/intqhc/mzac050

[6] Organisation WH. WHO Global Report on Falls Prevention in Older Age. vol. 2022. Switzerland: Geneva; 2007.

[7] Close JC, Lord SR. Fall assessment in older people. BMJ 2011;343. 10.1136/bmj.d5153 21917828

[8] Oliver D. Evidence for fall prevention in hospitals. J Am Geriatr Soc. 2008;56(9):1774–5. 19166460 10.1111/j.1532-5415.2008.01830.x

[9] Barker AL, Nitz JC, Low Choy NL, Haines T. Measuring fall risk and predicting who will fall: clinimetric properties of four fall risk assessment tools for residential aged care. Journals Gerontol Ser A: Biomedical Sci Med Sci. 2009;64(8):916–24. 10.1093/gerona/glp041 19414508

[10] Stapleton C, Hough P, Oldmeadow L, Bull K, Hill K, Greenwood K. Four-item fall risk screening tool for subacute and residential aged care: the first step in fall prevention. Australas J Ageing. 2009;28(3):139–43. 19845654 10.1111/j.1741-6612.2009.00375.x

[11] Perell KL, Nelson A, Goldman RL, Luther SL, Prieto-Lewis N, Rubenstein LZ. Fall risk assessment measures: an analytic review. Journals Gerontol Ser A: Biol Sci Med Sci. 2001;56(12):M761–6. 10.1093/gerona/56.12.m761 11723150

[12] Oliver D, Connelly JB, Victor CR, Shaw FE, Whitehead A, Genc Y, Vanoli A, Martin FC, Gosney MA. Strategies to prevent falls and fractures in hospitals and care homes and effect of cognitive impairment: systematic review and meta-analyses. BMJ. 2007;334(7584):82. 17158580 10.1136/bmj.39049.706493.55 PMC1767306

[13] Scott V, Votova K, Scanlan A, Close J. Multifactorial and functional mobility assessment tools for fall risk among older adults in community, home-support, long-term and acute care settings. Age Ageing. 2007;36(2):130–9. 17293604 10.1093/ageing/afl165

[14] Wabe N, Siette J, Seaman KL, Nguyen AD, Raban MZ, Close JC, Lord SR, Westbrook JI. The use and predictive performance of the Peninsula Health Falls Risk Assessment Tool (PH-FRAT) in 25 residential aged care facilities: a retrospective cohort study using routinely collected data. BMC Geriatr. 2022;22(1):1–11. 35365078 10.1186/s12877-022-02973-0 PMC8973529

[15] Wong Shee A, Phillips B, Hill K. Comparison of two fall risk assessment tools (FRATs) targeting falls prevention in sub-acute care. Arch Gerontol Geriatr. 2012;55(3):653–9. 22658287 10.1016/j.archger.2012.05.003

[16] Whitney J, Close JC, Lord SR, Jackson SH. Identification of high risk fallers among older people living in residential care facilities: a simple screen based on easily collectable measures. Arch Gerontol Geriatr. 2012;55(3):690–5. 22770712 10.1016/j.archger.2012.05.010

[17] Morley JE, Rolland Y, Tolson D, Vellas B. Increasing awareness of the factors producing falls: the mini falls assessment. J Am Med Dir Assoc. 2012;13(2):87–90. 22172397 10.1016/j.jamda.2011.11.002

[18] Australian Commission on Safety and Quality in Health Care. Preventing falls and harm from falls in older people. Best practice guidelines for Australian hospitals and residential aged care facilities. Canberra, Australia; 2009.

[19] Montero-Odasso M, van der Velde N, Martin FC, Petrovic M, Tan MP, Ryg J, Aguilar-Navarro S, Alexander NB, Becker C, Blain H. World guidelines for falls prevention and management for older adults: a global initiative. Age Ageing. 2022;51(9):afac205. 36178003 10.1093/ageing/afac205 PMC9523684

[20] Oliver D. Falls risk-prediction tools for hospital inpatients. Time to put them to bed? Age Ageing. 2008;37(3):248–50. 18456789 10.1093/ageing/afn088

[21] Ivankova NV, Creswell JW, Stick SL. Using mixed-methods sequential explanatory design: from theory to practice. Field Methods. 2006;18(1):3–20.

[22] The Peninsula Health Falls Prevention Services. Falls Risk Assessment Tool (FRAT) Instructions 2003. [https://training.aacs.com.au/wp-content/uploads/2016/08/Falls-Risk-Assessment-Tool-FRAT.pdf

[23] Department of Health and Ageing AG. Don’t fall for it. A guide to preventing falls for older people. Edited by Ageing DoHa. Canberra, Australia: Department of Health and Ageing, Australian Government; 2011.

[24] Kerse N, Butler M, Robinson E, Todd M. Fall prevention in residential care: a cluster, randomized, controlled trial. J Am Geriatr Soc. 2004;52(4):524–31. 15066066 10.1111/j.1532-5415.2004.52157.x

[25] Vieira ER, Palmer RC, Chaves PH. Prevention of falls in older people living in the community. BMJ 2016;353. 10.1136/bmj.i1419 27125497

[26] Thomas DR. A general inductive approach for analyzing qualitative evaluation data. Am J Evaluation. 2006;27(2):237–46.

[27] Braun V, Clarke V. Reflecting on reflexive thematic analysis. Qualitative Res Sport Exerc Health. 2019;11(4):589–97.

[28] Brown JM, Alverson EM, Pepa CA. The influence of a baccalaureate program on traditional, RN-BSN, and accelerated students’ critical thinking abilities. Holist Nurs Pract. 2001;15(3):4–8. 12120110 10.1097/00004650-200104000-00004

[29] Chabeli M. Facilitating critical thinking within the nursing process framework: a literature review. J Interdisciplinary Health Sci. 2007;12(4):69–89.

[30] Mangena A, Chabeli MM. Strategies to overcome obstacles in the facilitation of critical thinking in nursing education. Nurse Educ Today. 2005;25(4):291–8. 15896414 10.1016/j.nedt.2005.01.012

[31] Leonard M, Graham S, Bonacum D. The human factor: the critical importance of effective teamwork and communication in providing safe care. BMJ Qual Saf. 2004;13(suppl 1):i85–90. 10.1136/qshc.2004.010033 PMC1765783 15465961

[32] Rosenzweig M, Hravnak M, Magdic K, Beach M, Clifton M, Arnold R. Patient communication simulation laboratory for students in an acute care nurse practitioner program. Am J Crit Care. 2008;17(4):364–72. 18593836

[33] Waldron N, Hill A-M, Barker A. Falls prevention in older adults: assessment and management. Aus Fam Physician. 2012;41(12):930–5. 23210114

[34] Hopewell S, Adedire O, Copsey BJ, Boniface GJ, Sherrington C, Clemson L, Close JC, Lamb SE. Multifactorial and multiple component interventions for preventing falls in older people living in the community. *Cochrane Database of Systematic Reviews* 2018(7). 10.1002/14651858.CD012221.pub2 PMC6513234 30035305

[35] Gillespie LD, Robertson MC, Gillespie WJ, Sherrington C, Gates S, Clemson L, Lamb SE. Interventions for preventing falls in older people living in the community. Cochrane Database Syst Reviews 2012;9. 10.1002/14651858.CD007146.pub3 PMC8095069 22972103

[36] Panel on Prevention of Falls in Older Persons AGSaBGS. Summary of the updated American Geriatrics Society/British Geriatrics Society clinical practice guideline for prevention of falls in older persons. J Am Geriatr Soc. 2011;59(1):148–57. 21226685 10.1111/j.1532-5415.2010.03234.x

[37] Oliver D, Healey F, Haines TP. Preventing falls and fall-related injuries in hospitals. Clin Geriatr Med. 2010;26(4):645–92. 20934615 10.1016/j.cger.2010.06.005

[38] Barker A, Kamar J, Tyndall T, Hill K. Reducing serious fall-related injuries in acute hospitals: are low-low beds a critical success factor? J Adv Nurs. 2013;69(1):112–21. 22458341 10.1111/j.1365-2648.2012.05997.x

[39] Cortés OL, Piñeros H, Aya PA, Sarmiento J, Arévalo I. Systematic review and meta-analysis of clinical trials: in-hospital use of sensors for prevention of falls. Med (Baltim). 2021;100(41):e27467. 10.1097/MD.0000000000027467 PMC8519232 34731123

[40] Ming Y, Zecevic AA, Hunter SW, Miao W, Tirona RG. Medication review in preventing older adults’ fall-related injury: a systematic review and meta-analysis. Can Geriatr J. 2021;24(3):237–50. 34484506 10.5770/cgj.24.478 PMC8390322

[41] Seppala LJ, Kamkar N, van Poelgeest EP, Thomsen K, Daams JG, Ryg J, Masud T, Montero-Odasso M, Hartikainen S, Petrovic M, van der Velde N. Medication reviews and deprescribing as a single intervention in falls prevention: a systematic review and meta-analysis. Age Ageing 2022;51(9). 10.1093/ageing/afac191 PMC9509688 36153749

[42] Oliver D, Daly F, Martin FC, McMurdo MET. Risk factors and risk assessment tools for falls in hospital in-patients: a systematic review. Age Ageing. 2004;33(2):122–30. 14960426 10.1093/ageing/afh017

[43] Seidi J, Alhani F, Salsali M. Nurses’ clinical judgment development: a qualitative research in Iran. Iran Red Crescent Med J 2015;17(9). 10.5812/ircmj.20596 PMC4601210 26473075

[44] Tanner CA. Thinking like a nurse: a research-based model of clinical judgment in nursing. J Nurs Educ. 2006;45(6):204–11. 16780008 10.3928/01484834-20060601-04

[45] Braithwaite J, Wears RL, Hollnagel E. Resilient health care, volume 3: reconciling work-as-imagined and work-as-done. Boca Raton, FL: CRC; 2016.

[46] Malina MA, Nørreklit HS, Selto FH. Lessons learned: advantages and disadvantages of mixed method research. Qualitative Res Acc Manage. 2011;8(1):59–71.

