# Supplementary material for: The use and usefulness of the Peninsula Health Falls Risk Assessment Tool (PHFRAT) process in residential aged care: a mixed methods study across 25 aged care facilities
Source: BMC Geriatr. 2024 Oct 24;24:869. doi: 10.1186/s12877-024-05462-8 (PMC11515602; doi:10.1186/s12877-024-05462-8)
Supplement: Supplementary file 1 — Supplementary Material 1 [file 12877_2024_5462_MOESM1_ESM.docx]

# Appendixes

## Appendix 1: Procedures of FRAT assessment

PHFRAT consists of three components, i.e., part 1, part 2 and part 3. Part 1 assesses residents’ level of fall risk by evaluating four risk factors (i.e., recent falls, medication use, psychological status, and cognitive status) and two specific health problems (i.e., whether having recent change in functional status and/or medications affecting safe mobility and whether having dizziness or postural hypotension). The status of four fall risk factors is categorised into four levels scored differently from 1 to 6 (Table 1), with a total score of 5-11 considered low fall risk, 12-15 as medium risk, and 16-20 as high risk. Residents reporting any of the two specific health problems were automatically classified as high risk.

Table A1-1 Fall risk factors and the scoring method

| **Risk factor** | **Level** | **Risk score** |
| --- | --- | --- |
| **Recent falls** | 0 in last 12 months | 2 |
|  | ≥ 1 between 3–12 months ago | 4 |
|  | ≥ 1 in last 3 months | 5 |
|  | ≥ 1 in last 3 months whilst inpatient/resident | 6 |
| **Medication use**  (Sedatives; Anti-Depressants; Anti-Parkinson’s; Diuretics; Anti-Hypertensives) | not taking any of these | 1 |
|  | taking 1 | 2 |
|  | taking 2 | 3 |
|  | taking 3 or more | 4 |
| Psychological status  (Anxiety; Depression; Cooperation, Insight or Judgement esp, re mobility) | does not appear to have any of these | 1 |
|  | appears mildly affected by one or more | 2 |
|  | appears moderately affected by one or more | 3 |
|  | appears severely affected by one or more | 4 |
| **Cognitive status**  (Hodkinson Abbreviated Mental Test Score (AMTS) or Mini–Mental State Examination [MMSE]) | score 9-10/10 **OR** intact | 1 |
|  | score 7–8 mildly impaired | 2 |
|  | score 5–6 moderately impaired | 3 |
|  | score 4 or less severely impaired | 4 |

Part 2 assesses ten fall risk factors at both individual and environmental levels. The first nine risk factors of falls are related to residents’ problems of: vision, mobility, transfers, behaviour, activity of daily living (ADL), environment, nutrition, continence, and others, as detailed in Table 2. Regarding the tenth fall risk factor—fall history, part 2 not only documents whether residents had falls previously and also records the corresponding time, locations, and causes of previous four falls. Part 2 also provides detailed descriptions of residents’ fall-related problems in a free-text format to assist understand residents’ vulnerability to falls.

Table A1-2 Checklist of fall risk factors

| **Risk factors** | **Description** |  |
| --- | --- | --- |
| Vision | Reports/observed difficulty seeing—objects / finding way around/signs | **□** |
| Mobility | Mobility status unknown or appears unsafe / impulsive / forgets gait aid | **□** |
| Transfers | Transfer status unknown or appears unsafe i.e. over-reaches, impulsive | **□** |
| Behaviours | Observed or reported agitation, confusion, disorientation Difficulty following instructions or non-compliant (observed or known) | **□** |
| ADL | Observed risk-taking behaviours, or reported from donor facility  Observed unsafe use of equipment  Unsafe footwear / inappropriate clothing | **□**  **□**  **□** |
| Environment | Difficulties with orientation to environment i.e. areas b/w bed/bathroom / dining room | **□** |
| Nutrition | Underweight / low appetite | **□** |
| Continence | Reported or known urgency / nocturia / accidents | **□** |
| Other | Osteoporosis, history fractures | **□** |

Part 3 records the fall interventions that residents received. These fall interventions are developed based on assessors’ experience or built on existing fall intervention strategies. Part 3 is documented in a free-text format and is updated each time when a new PHFRAT assessment is conducted. After part 3, PHFRAT ends up with a planned review date set by the assessors for another PHFRAT assessment.

## Appendix 2: Interview schedule

**Research question:** Understating/identifying the mechanisms of how falls risk assessment tool-related information is utilised to inform fall prevention in RAC facilities: A mixed-method study.

**Pre-script:**

Hi, my name is [name] and I’m a [job title] at the Australian Institute of Health Innovation at Macquarie University. I am part of a research team at Macquarie University that is working with Anglicare to analyse falls and falls management in aged care. This project includes many research activities, some of which you may have heard about at your facility. In today’s interview, we aim to explore how the PHFRAT assessment is used in residential aged care.

This interview is structured so that first we discuss how the PHFRAT assessment is used in your facility. After this, I want to hear your opinion on some results we have found in an analysis of the PHFRAT assessment.

There are no right or wrong answers in this interview. The interview is audio-recorded so that the valuable information you provide can be studied in detail. During the interview, we will be on a first-name basis, but we won’t be using your name in any future research documents or publications.

All data we collect will be treated as confidential. It will be deidentified using a code and securely stored. Any information you provide will not be shared with anyone outside the research team.

Participation in this project is entirely voluntary. You can abandon the interview at any time without consequence. Participation in this interview will not impact your relationship with any organisations.

**Q1. What position do you have? e.g., nurse, physio**

**Q2. How long have you been working at this facility?**

**Q3. How many PHFRAT assessments have you done before?**

**(The answer can be in certain range, e.g., 5-10, rather than an exact number. Or, we can provide some options, e.g., A 0-10, B 11-50, C 50-99, D ≥ 100).**

**Asking the number of PHFRAT assessments done before might be not appropriate because the staff can do many PHFRAT assessments at one time. In this case, how about asking ‘how many times of PHFRAT assessments you have done before? ’**

**PHFRAT Interviews Question Guide**

***Work-as-imagined***

*[present work as imagined diagram]*

This is a process map of the PHFRAT tool. It includes **PHFRAT A (risk level), B (risk factor checklist), C (strategies)**, and a flowchart of the PHFRAT review process.

**Questions:**

1. Do you think that this process map accurately describes how PHFRAT assessments are done in your facility? Are there any procedures missing from this map?
2. Who completes PHFRAT A, B, and C?
3. Does PHFRAT A (risk level) & B (risk factor checklist) guide PHFRAT C?
4. Who completes personal/specific conditions? e.g., requesting for bedrails, resident’s preference to drink alcohol.
5. Who fills out the review date? How do you/they do it?
6. Are the results of the PHFRAT assessment handed over to the rest of the care team? How?
7. What do you think about the PHFRAT assessment?
   1. Is the information entered in them accurately?
   2. Do the interventions identified in PHFRAT C improve fall management in your facility?
   3. Would you change the PHFRAT assessment?

***Work-as-done***

This is the same process map of the PHFRAT tool. But this time we have added numbers/percentages to the PHFRAT components. The numbers represent the number of residents who had that component of the PHFRAT. For example, if you look at PHFRAT A, 26% of residents were classified as low risk, 40% as medium risk, and 34% as high risk, out of 703 PHFRATs in 2019**.**

**Questions:**

1. In our analysis we found that 10% of PHFRATs have **personal/specific conditions** added to the assessment. What do you think would trigger yourself/someone else to complete this section?
2. We also found 88% of the total PHFRATs had strategies in place (e.g., place buzzer close to resident’s reach always, initiate toileting routine), why do you think some PHFRATs have no strategies in place?
3. When we took a closer look at the strategies in PHFRAT C, we found that there was little difference in the number or type of strategies recommended to low, medium, and high-risk residents. Why do you think that the strategies recommended to residents are similar despite their risk status?
4. In our analysis, we found that 78% of PHFRATs are missing review dates. If a resident does not have a fall within six months, when would the PHFRAT be updated?
5. When the PHFRAT is reviewed, do you think that PHFRAT A, B, and C are accurately updated?

**Final thoughts:**

Is there anything you want to add about the PHFRAT assessment in residential aged care?

**Concluding statement**

Thank you for participating in this interview. If you need to contact us again about your participation, please use the contact details provided in the participant information sheet.

## Appendix 3: Types of fall prevention/intervention measures recorded in PHFRAT part 3 by fall risk factor identified in part 2 and fall risk level assessed in part 1


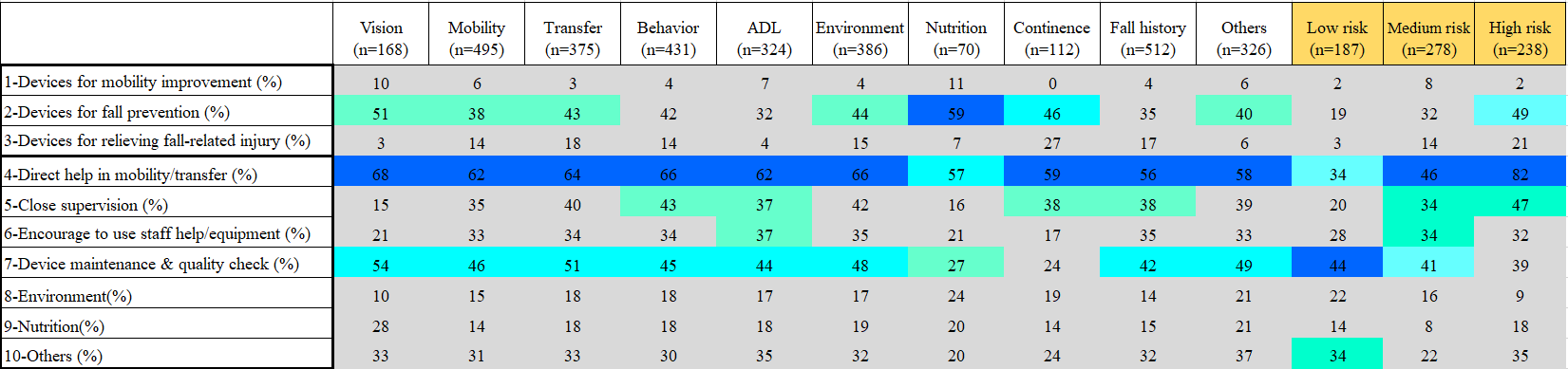


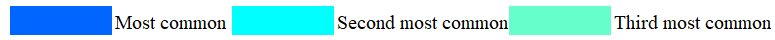


*Note: there might be multiple fall intervention measures for one fall risk factor. Thus, the sum of the proportions for each fall risk factor might exceed 100%.*
